# Cytogenetic studies in three octopods, *Octopusminor*, *Amphioctopusfangsiao*, and *Cistopuschinensis* from the coast of China

**DOI:** 10.3897/CompCytogen.v12i3.25462

**Published:** 2018-09-04

**Authors:** Jin-hai Wang, Xiao-dong Zheng

**Affiliations:** 1 Institute of Evolution and Marine Biodiversity, Ocean University of China, Qingdao 266003, China Ocean University of China Qingdao China; 2 Key Laboratory of Mariculture, Ocean University of China, Ministry of Education, China Ocean University of China Qingdao China

**Keywords:** octopods, karyotype, Ag-NORs, C-bands, genome size, flow cytometry

## Abstract

To provide markers to identify chromosomes in the genome of octopods, chromosomes of three octopus species were subjected to NOR/C-banding. In addition, we examined their genome size (*C* value) to submit it to the Animal Genome Size Database. Silver staining revealed that the number of Ag-nucleoli was 2 (*Octopusminor* (Sasaki, 1920)), 2 (*Amphioctopusfangsiao* (d’Orbigny, 1839)) and 1 (*Cistopuschinensis* Zheng et al., 2012), respectively, and the number of Ag-nucleoli visible was the same as that of Ag-NORs on metaphase plates in the same species. In all analyzed metaphases, Ag-NORs were mainly located terminally on the long arms of chromosomes 3 (3^rd^) of *O.minor* and on the short arms of chromosomes 4 (4^th^) of *A.fangsiao*, whereas only one of the chromosomes 23 (23^rd^) was found Ag-NORs of *C.chinensis*. C-bands were localized predominantly in the centromeric regions of chromosomes in the three species, while other conspicuous stable C-bands were observed in terminal regions, including the Ag-NORs. That means these three chromosome pairs (3^rd^, 4^th^ and 23^rd^) could be considered species-specific cytogenetic markers. The mean *C* values of *O.minor*, *A.fangsiao* and *C.chinensis* were 7.81±0.39 pg (0.070 pg per unit length), 8.31±0.18 pg (0.068 pg per unit length) and 5.29±0.10 pg (0.038 pg per unit length), respectively, and results showed that *C* values of the three species were not proportional to the relative length of the chromosomes. These cytogenetic characteristics will provide more theoretical foundation for further researches on chromosome evolution in octopods.

## Introduction

Genetics and cytology combine to establish cytogenetics, mainly from the perspective of cytology, especially from a chromosome structure and function as well as the relationship between chromosomes and other organelles, to elucidate the mechanism of inheritance and variation. Cytogenetic analysis usually includes chromosome karyotype, band type, flow karyotype analysis and fluorescence *in situ* hybridization. Previous published reviews on chromosomal studies of molluscs were greatly increased since cytogenetic techniques including silver-staining, C-and G-banding and have begun to be applied to molluscan chromosomes (Thiriot-Quiévreux 2003). Although these techniques have been widely used in the study of shellfish chromosomes, there are no reports on the cephalopods.

Octopods, such as *Octopusminor* (Sasaki, 1920), *Amphioctopusfangsiao* (d’Orbigny, 1839) and *Cistopuschinensis* Zheng et al., 2012 are cephalopod species. The previous chromosome analysis of cephalopods had revealed 2n=60 or 92 in most species studied, including *O.minor*, *A.fangsiao*, *C.chinensis*, two sepiids (*Sepiaesculenta* and *S.lycidas* Gray, 1849) and three loliginids (*Heterololigobleekeri* Natsukari, 1984, *Sepioteuthislessoniana* Blainville, 1824 and *Photololigoedulis* (Hoyle, 1885)) (Gao and Natsukari 1990, Adachi et al. 2014, Wang and Zheng 2017), although *Nautilusmacromphalus* Sowerby, 1849 had 2n=52 chromosomes (Bonnaud et al. 2004), and the studies led by Papan and Jazayeri reported the chromosome number of *S.arabica* Massy, 1916 and *S.pharaonis* Ehrenberg, 1831 was 2n=48 (Papan et al. 2010, Jazayeri et al. 2011). However, there are only a few studies on the cephalopod chromosomes in reported publications, and there are no researches on the band type in these species. Adachi et al. (2014) first tried to use fluorescence *in situ* hybridization on the cephalopod chromosomes and suggested that the telomere sequence of *O.areolatus* de Haan, 1839–1841 was (TTAGGG)n, but there was a lack of complete and clear metaphases in the report. Due to the restriction of the embryo acquisition, and the number of cephalopod chromosomes up to 60, it is difficult to obtain an ideal metaphase. All these factors seriously restrict the cytological study of cephalopods. In a previous published paper, we gave a detailed overview of the existing cephalopod chromosome information, including the genetic relationship analysis based on evolutionary distance (Wang and Zheng 2017). The present study used gills as materials, and through a large number of repeated tests, the ideal metaphases with NORs and C-bands were obtained based on the previous study.

As an important part of the study of cytogenetics, more and more genome sizes (*C* values) have been revealed. Although the genome sizes of 281 mollusks have been submitted to the Animal Genome Size Database (http://www.genomesize.com. Accessed December 25, 2017) while there just have been 6 species of cephalopod *C* values that can be obtained from the database: *O.bimaculatus* (Hinegardner 1974), *O.bimaculoides* (Albertin et al. 2015), *O.vulgaris* (Packard and Albergoni 1970), *Euprymnascolopes* (Adachi et al. 2014), *Loligoplei* (Hinegardner 1974) and Loliginidae sp. (Mirsky and Ris 1951). These *C* values were estimated based on bulk fluorometric assay and feulgen image analysis densitometry. As genomic and transcriptomic sequencing is carried out in cephalopods, more and more cephalopod genome sizes have been revealed by complete genome sequencing such as *N.pompilius*, *Architeuthisdux*, *Hapalochlaenamaculosa*, *E.scolopes*, *Idiosepiusparadoxus*, *L.pealeii*, *S.officinalis*, etc (Yoshida et al. 2011, Albertin et al. 2012) (Table 1). Besides, Adachi et al. (2014) examined the *C* values of *O.ocellatus* and *O.vulgaris* based on flow cytometry. Although there are many methods to detect *C* values, we choose flow cytometry for the convenience, rapid analysis and relative accuracy of the sample preparation (Gokhman et al. 2017).

To develop octopus chromosome markers, the present research has first completed NOR/C-banding. Also we examined their *C* values to submit to the Animal Genome Size Database. This is a basic work for molecular cytogenetic research of octopods. It is expected to lay a solid theoretical foundation for further researches on chromosome evolution in octopods.

**Table 1.** The published information of cephalopod genome size. **BA**: Biochemical Analysis, **FCM**: Flow Cytometry, **BFA**: Bulk Fluorometric Assay, **CGS**: Complete Genome Sequencing, **FIA**: Feulgen Image Analysis Densitometry.

Species	Origin	Internal standard	Method	*C* value (pg)/genome size (Gb)	References
* O. vulgaris *	sperm	Not specified	BA/CGS	5.15 pg/2.5–5 Gb	Packard and Albergoni 1970; Albertin et al. 2012
* O. minor *	Haemocytes	* G. domesticus *	FCM	7.82±0.56 pg	This study
* O. bimaculatus *	sperm	* Strongylocentrotus purpuratus *	BFA	4.30 pg	Hinegardner 1974
* O. bimaculoides *	Not specified	Not specified	BFA/CGS	2.93 pg/3.2 Gb	Albertin et al. 2012, 2015
* A. fangsiao *	Haemocytes	* G. domesticus *	FCM	8.23±0.42 pg	This study
* C. chinensis *	Haemocytes	* G. domesticus *	FCM	5.13±0.38 pg	This study
* H. maculosa *	–	–	CGS	4.5 Gb	Albertin et al. 2012
* S. officinalis *	–	–	CGS	4.5 Gb	Albertin et al. 2012
* L. plei *	sperm	* S. purpuratus *	BFA	2.80 pg	Hinegardner 1974
* L. pealeii *	–	–	CGS	2.7 Gb	Albertin et al. 2012
* E. scolopes *	Haemocytes, Sperm	* G. domesticus *	FIA/CGS	3.75 pg/3.7 Gb	Gregory 2013; Albertin et al. 2012
* I. paradoxus *	–	–	CGS	2.1 Gb	Yoshida et al. 2011
* A. dux *	–	–	CGS	4.5 Gb	Albertin et al. 2012
* N. pompilius *	–	–	CGS	2.8–4.2 Gb	Yoshida et al. 2011

## Material and methods

### Ag-staining of the NORs and C-bands

Collection of samples and chromosome slides preparation based on the previous published paper (Wang and Zheng 2017). The nucleoli in interphase and the NORs in metaphase were visualized using rapid silver nitrate staining using the previous methods (Howell and Black 1980). C banding were carried out following the protocols of Sumner (1972) with some modifications. The dyed chromosome slides were detected under a light microscope with an oil lens (Leica MC170 HD, Germany).

### Genome size (*C* value)

**Preparation of cell suspension**

Ten individuals (5 males and 5 females) of each species were used for collecting hemolymph. All subjects were handled according to the guidelines put forth by the EU Directive 2010/63/EU for cephalopod welfare (Fiorito et al. 2014). Before dissecting, all octopods should be anesthetized using 7.5% magnesium chloride (MgCl_2_) solution (Messenger et al. 1985) until unconscious.

Then hemolymph was withdrawn from the heart or cephalic aorta of each octopus using a disposable syringe, and the hemolymph was immediately transferred into a 1.5 ml centrifuge tube containing precooled (4 °C) phosphate-buffered saline (PBS) (v/v=1:1). Mixed samples were centrifuged for 12 min at 300g and 4 °C, and then the hemocytes were resuspended twice in PBS according to above. The last suspension containing 300 μl PBS was added to another tube containing 900 μl precooled (–20 °C) anhydrous ethanol (v/v=1:3), fixing at least 3 hours at 4 °C. The fixed cell was washed twice in PBS after it was suspended with PBS up to 1 ml. Before the flow cytometry detection, a moderate propidium iodide solution (PI, 20–30 μg/ml) was added to the suspensions, staining for 2 hours at 4 °C in dark.

**Flow cytometry analysis**

Previous studies have shown that the genome size (*C* value) of chickens (*Gallusdomesticus*) was 1.25 pg (Tiersch et al. 1989, Adachi and Okumura 2012, Adachi et al. 2014). Here we determined *C* values of octopods using this chicken red blood cells as internal standard, and the standard sample was purchased from BD company (DNA QC Particles).

*C* value was measured using a model PA flow cytometer (Beckman Coulter Cytomics FC 500 MPL), in principle, at least 15,000 cells were measured in each sample. The blue light of 488 nm was first excited, and the fluorescence of PI was detected by the emission wave length of 625±10 nm. The present study used chicken standard sample as calibration instrument, and then used it as the internal standard, by comparing multiple relationships between the standard sample (chicken red blood cells) and the pending sample (octopus hemolymph) peak, calculating the *C* values.

## Results

### Banding analysis

**Figure 1.**
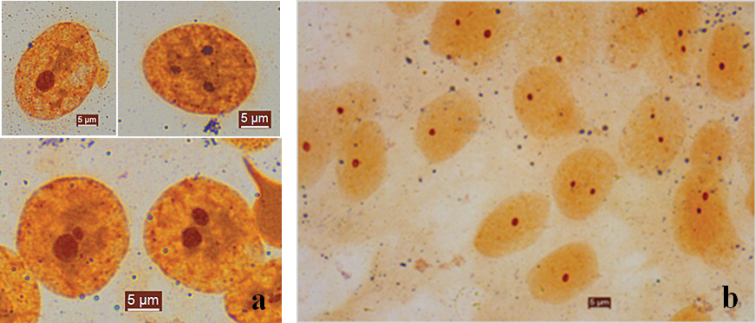
Ag-nucleoli of interphase. **a** 1, 2 or 3 normal nucleolus organizer regions (NORs) in interphase of *O.minor* and *A.fangsiao*, and the two species mainly contain 2 NORs**b** 1 or 2 NORs in interphase of *C.chinensis*, and most of them contain 1 NORs . Scale bar: 5 μm.

**Figure 2.**
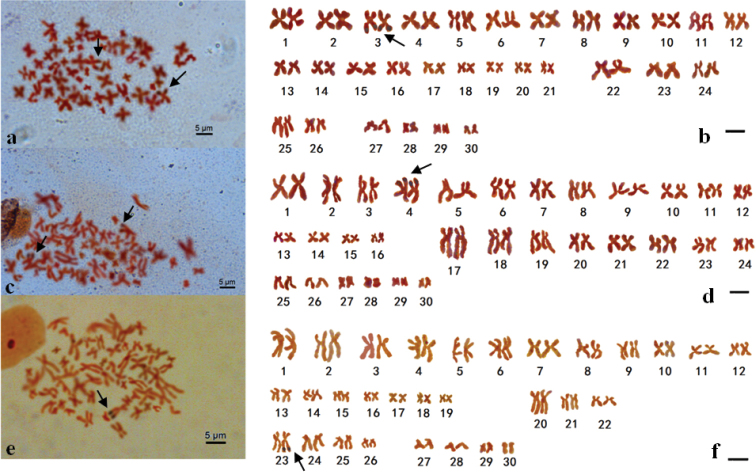
Karyotypes and NOR-bearing chromosomes from three species of octopod gills. **a** The metaphase plate of *O.minor***b** Karyogram of *O.minor* from (**a**) showed that Ag-NOR loci were located on the long arms of metacentric chromosome 3 **c** The metaphase plate of *A.fangsiao***d** Karyogram of *A.fangsiao* from (**c**) showed that Ag-NOR loci were located on the short arms of metacentric chromosome 4 **e** The metaphase plate of *C.chinensis***f** Karyogram of *C.chinensis* from (**e**) showed that Ag-NOR loci were located on long arms of subtelomeric chromosome 23. Arrows indicate the NOR-bearing chromosomes. Scale bars: 5 μm.

After silver staining was performed, the number of Ag-nucleoli was between 1–3 in interphase nuclei of three species (Fig. 1). We randomly selected 200 interphase nuclei to calculate the number of nucleolar organizer regions for each species. Among the scored interphases 24% had 1 nucleolus, and 61% had 2 nucleoli, and 10% had 3 nucleoli and 5% had more than 3 nucleoli in *O.minor* species. Twenty-four metaphases were counted indicating there were 2 Ag-NORs, then 7 of them were selected for karyotype analysis and Ag-NOR loci were located on the long arms of metacentric chromosomes 3 (3^rd^). In *A.fangsiao* species, 38 of 200 interphase nuclei showed 1 Ag-nucleolus, then 146 of them contained 2 nucleoli and 16 of them had 3 to 5 nucleoli. Eighteen metaphases were counted and 7 of them were selected for karyotype analysis, showing there were 2 Ag-NORs and Ag-NOR loci were located on the short arms of the metacentric chromosomes 4 (4^th^). For *C.chinensis* species, there were mainly 1 nucleolus (up to 68%), followed by 2 nucleoli accounting for about 21%, while a small proportion had 3–5 nucleoli. Selected 13 scored metaphases indicated that there were only 1 Ag-NOR and then 7 karyotypes were analyzed suggesting that Ag-NOR loci were located on long arms of the subtelomeric chromosome 23 (23^rd^) (Fig. 2).

**Figure 3.**
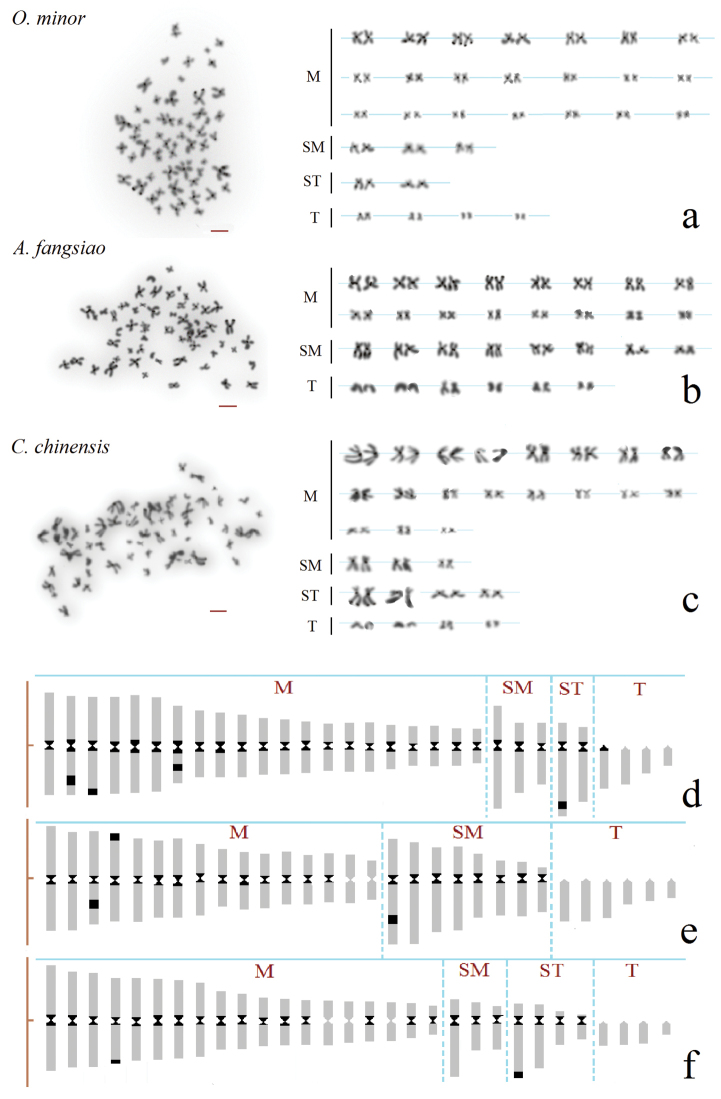
C-banding karyotypes arranged from mitotic metaphases of *O.minor* (**a**), *A.fangsiao* (**b**) and *C.chinensis* (**c**) and diagrams of C-banding (**d, e, f**). Black dots representing the chromosomes with C bands/heterochromatin blocks. Scale bars: 5 µm.

The C-band karyotype analysis indicated that there were 31 C-bands (*O.minor*), 25 C-bands (*A.fangsiao*) and 25 C-bands (*C.chinensis*) in three species of octopods, respectively. C-bands were consistently localized in the centromeric regions of most chromosomes in the three species, but which varied in size (Fig. 3d–f), and in *C.chinensis*, the C-bands of long arms were smaller those of *O.minor* and *A.fangsiao*. In particular, several other steady C-bands were located on terminal region of chromosomes 3 (3^rd^) for *O.minor*, chromosomes 4 (4^th^) for *A.fangsiao* and chromosomes 23 (23^rd^) for *C.chinensis*, which was coincident with positive Ag-NOR loci, except for chromosomes 23 in *C.chinensis*, where only one of the chromosomes 23 was found to have an Ag-NOR (Fig. 2f). Besides, various C-bands were observed on the long arms of chromosome pairs 2 (proximal terminal region), 7 (interstitial region) and 25 (proximal terminal region) in *O.minor*, chromosome pairs 3 (interstitial region) and 17 (interstitial region) in *A.fangsiao* and chromosome pair 4 (terminal region) in *C.chinensis*.

Combined with the results of silver staining and C-banding, although C-bands were localized predominantly in the centromeric regions of chromosomes in the three octopus species, other conspicuous C-bands were observed in terminal regions, including the Ag-NORs. Therefore, we can select chromosome pairs 3, 4 and 23 as effective species-specific markers to distinguish the three octopods.

### *C* value

**Figure 4.**
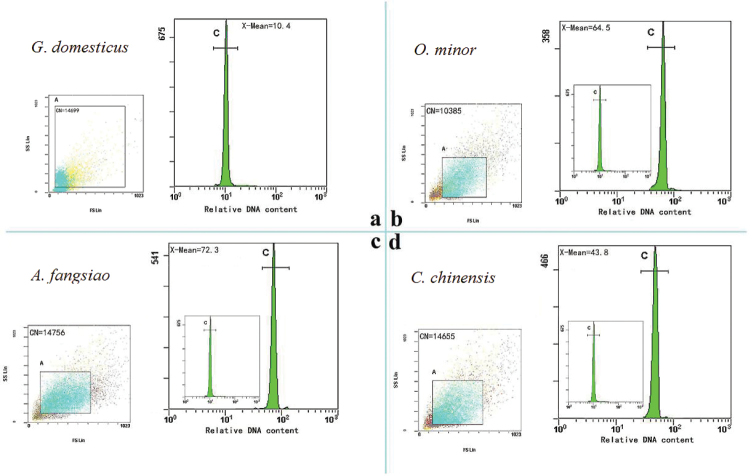
Flow cytometry profiles of relative fluorescence intensity of propidium iodide (PI) in octopus hemocytes of **b***O.minor***c***A.fangsiao* and **d***C.chinensis* with *G.domesticus***a** as standard (St). Scatter plots display the quality and number of cell mass of standard samples and test samples, and the peak figures show the relative DNA content of each sample. **b**, **c** and **d** are just a representative graph of the three species of octopus samples. CN, the number of cells; X-MEAN, mean fluorescence intensity.

In data analysis, we selected about 15,000 cells per sample, then fluorescence intensity representing the relative DNA content was recorded. Figure 4a showed the number of cells (CN=14699) and mean fluorescence intensity (X-Mean=10.4) of the internal standard and other three representative results of *O.minor* (CN=10385, X-Mean=64.5, Fig. 4b), *A.fangsiao* (CN=14756, X-Mean=72.3, Fig. 4c) and *C.chinensis* (CN=14655, X-Mean=43.8, Fig. 4d) also were enumerated to estimate the DNA content. The results showed the mean *C* values of *O.minor*, *A.fangsiao* and *C.chinensis* were 7.81±0.39 pg (male 7.85±0.47 pg, female 7.76±0.32 pg), 8.31±0.18 pg (male 8.33±0.25 pg, female 8.30±0.10 pg) and 5.29±0.10 pg (male 5.28±0.08 pg, female 5.29±0.12 pg), respectively. *C.chinensis* had the smallest *C* value, significantly lower than *O.minor* (*P* < 0.05) and *A.fangsiao* (*P* < 0.05) (Table 2).

**Table 2.** The results of *C* values from three species of octopods.

**Species**		**Sample no.**	**Fluorescence intensity**	***C* value/pg**	***C* value (X±SE) /pg**	
* G. domesticus *		0	10.4	1.25	1.25	
* O. minor *	male	1	63.5	7.63	7.85±0.47	7.81±0.39
		2	60.1	7.22		
		3	70.4	8.46		
		4	67.2	8.08		
		5	65.5	7.87		
	female	6	61.3	7.37	7.76±0.32	
		7	63.5	7.63		
		8	67.2	8.08		
		9	66.1	7.94		
* A. fangsiao *	male	1	72.3	8.69	8.33±0.25	8.31±0.18
		2	68.9	8.28		
		3	67.5	8.11		
		4	68.4	8.22		
	female	5	70.2	8.44	8.30±0.10	
		6	68.8	8.27		
		7	68.3	8.21		
		8	69.0	8.29		
* C. chinensis *	male	1	43.8	5.26	5.28±0.08	5.29±0.10
		2	44.6	5.36		
		3	43.3	5.20		
	female	4	43.5	5.23	5.29±0.12	
		5	44.8	5.38		
		6	45.0	5.41		
		7	42.9	5.16		

Based on our previous studies, the chromosome total relative lengths of *O.minor*, *A.fangsiao* and *C.chinensis* were 112.33, 122.77 and 139.20. *C.chinensis* had the largest relative length, followed by *A.fangsiao* and *O.minor*, which was not proportional to the *C* value. Obviously, *C.chinensis* had the smallest *C* value but the largest chromosome relative length. The DNA content of the unit length chromosomes of the three octopods was about 0.070 pg (*O.minor*), 0.068 pg (*A.fangsiao*) and 0.038 pg (*C.chinensis*) respectively. Results suggested that there was no significant positive proportional relationship between the *C* value and the relative chromosome length. Besides, this study analyzed the *C* values of 14 species of cephalopods, ranging of 2.20 to 8.23 pg (2.10–7.86 Gb), of which *I.paradoxus* had the smallest genome size and the largest value from *A.fangsiao*. Overall, the average genome size of 6 species from Octopoda (3.35–8.23 pg) was higher than that of 8 species from Sepiida and Teuthida (2.20–4.71 pg).

## Discussion

We first carried out silver staining (Ag-NOR) in octopus species, and the results showed that *C.chinensis* had only one nucleolus organizer region (NOR) which was located terminally at the long arms of a pair of homologous chromosomes. *O.minor* and *A.fangsiao* had two NORs which located terminally on a pair of homologous chromosomes. As an effective chromosome marker, polymorphisms in NORs can be observed in interspecies or intraspecies comparisons, including the number, location and size of sliver staining (Wang et al. 2015, Zaleśna et al. 2017), even the geographical location and habitat differences can cause the diversity. However, many studies have determined that the number of NORs in interphase is consistent with that on metaphase plates of the same species (Iizuka et al. 2013, Zaleśna et al. 2017). According to the report of Okumura et al. (1999), NORs of the *Haliotisdiscushannai* were located at the end of two pairs of chromosome long arms, and it was also found in the subcentral centromeric chromosome and the centromeric chromosome. Similarly, in the later studies of abalone chromosomes from Wang et al. (2015) showed NOR sites located on the 14^th^ and 17^th^ chromosomes, but at the end of the short arms of central and submetacentric chromosomes also were found the sites. From the two studies, the same species proved that the polymorphism of Ag-NOR bands was prevalent among most species, including crustaceans, teleost fish, reptiles, mammals and other mollusks (Babu and Verma 1985, Thiriot-Quiévreux and Insua 1992, Vitturi and Lafargue 1992, Cross et al. 2003, Britton-Davidian et al. 2011).

The number of C-bands in *O.minor* was larger than that of the other two octopus species, which was consistent with the Ag-NORs. Although *A.fangsiao* and *C.chinensis* had the same number of C-bands, while the former contained more interstitial C-bands. Taking these two points into account, it is confirmed that the *O.minor* is more advanced in evolution from the chromosome level. Almost all chromosomes of three species of octopods can show C-bands in centromere regions, and it means that heterochromatic blocks are evident in the pericentromeric regions of chromosomes, which is consistent with the traditional view that the centromere region is mainly consisted of heterochromatin. The C-banding results showed that the NOR regions of the three species were all deep-stained C-bands, which also coincided with the common assumption that the NOR regions were composed of heterochromatin. The stable C-bands can be used as markers for chromosome identification, while the higher polymorphic C-band is not suitable as a marker for distinguishing chromosomes, but it can be used as a genetic marker for the study of chromosome polymorphism. In present study, chromosomal markers for identifying three species of octopods were developed by Ag-NORs and C-bands, and it was effective means. Long before that, Martinez-Lage et al. (1995) managed to separate the chromosomes of three shellfish by banding techniques, which confirmed the reliability of this method.

In the present study, due to different survival pressure from geographical distribution (Zhang and Onozato 2004), the genome size of *C.chinensis* from South is significantly less than that of *O.minor* and *A.fangsiao* from North. Adachi et al. (2014) pointed out that the genome size of O. (A.) areolatus was 5.47 pg, then this study showed the value was about 8.23 pg, significantly higher than the former. The main reason may be the difference of samples or the existence of hidden species. Although some studies had shown (Rakic et al. 2014) that the genome size was related to ecological factors, that was not absolute. The diversity of genome size involved the interaction of multiple factors and can not simply attribute the differences to the external environmental factors.

DNA is linear on the chromosome. According to Adachi et al. (2014), genome size of O. (A.) areolatus and *O.vulgaris* was proportional to the relative chromosome length, they determined the values of the two octopods was 5.47 pg and 3.50 pg, respectively. The genome size of O. (A.) areolatus was about 1.5 times higher than that of *O.vulgaris*, and this ratio coincided with the ratio of chromosome length to 122.60/66.30. Even so, we can not simply consider the existence of ploidy between the two, because the number of chromosomes is identical. Therefore, we speculate that genome duplication may occur during the evolution of *O.vulgaris*, which leads to the ploidy relationship. In contrast, current studies have found that the genome size of *O.minor*, *A.fangsiao* and *C.chinensis* have no obvious linear relationship with their chromosome length. Different methods to detect the genome size of the same species usually yield different results. In the reported cephalopod genomes, the genome size of *O.vulgaris*, *O.bimaculoides*, and *E.scolopes* have been determined by biochemical analysis, bulk fluorometric assay, complete genome sequencing and feulgen image analysis densitometry (Table 1). Regardless of the cephalopod species, the obtained genome size by complete genome sequencing is generally larger than other testing methods. For example, using biochemical analysis method to get *O.vulgaris* genome size is 5.15 pg (Packard and Albergoni 1970), and the result of genome sequencing is 2.5 to 5.0 Gb, about 2.62 to 5.24 pg (Albertin et al. 2012). The *O.bimaculoides* genome size is 2.93 pg/3.2 Gb (about 3.35 pg) by bulk fluorometric assay (Albertin et al. 2012) and genome sequencing (Albertin et al. 2015), respectively. Besides, the *E.scolopes* genome size by feulgen image analysis (3.75 pg) (Gregory 2013) is less than that of genome sequencing (3.7 Gb, about 3.87 pg) (Albertin et al. 2012). The main reason for these results is that genome size obtained by genome sequencing contains a complete set of nucleotide sequences, including non-coding sequences, and the increase of non-coding sequences largely obscures the correlation between genome size and species evolution complexity. In addition, the statistical analysis of cephalopod genome size is mainly based on the existing basic data. More cephalopod genomes are needed to be sequenced to further analyze the genomic characteristics of the population.

In conclusion, the present study combining a previously published paper (Wang and Zheng 2017) highlights our increased knowledge of cephalopod cytogenetic studies. Up to now, cytogenetic studies of the cephalopods have stepped forward: Thirteen species of cephalopod chromosome information have been reported, of which three are related to silver staining and C-banding, also fourteen species of cephalopod genome size or haploid DNA content have been revealed. What needs to be done next is the location of the functional genes (such as sex related genes) on the chromosomes to further deepen cytogenetic study of cephalopods.
